# Infants needed to immunise with nirsevimab to prevent one RSV hospitalisation, Spain, 2023/24 season

**DOI:** 10.2807/1560-7917.ES.2025.30.6.2500040

**Published:** 2025-02-13

**Authors:** Roberto Pastor-Barriuso, Olivier Núñez, Susana Monge, the Nirsevimab Effectiveness Study Collaborators, The Nirsevimab Effectiveness Study Collaborators, Carmen Olmedo, David Moreno-Perez, Nicola Lorusso, Sergio Fernández Martínez, Pedro Eliseo Pastor Villalba, María Ángeles Gutierrez Rodriguez, Marcos Alonso Garcia, Pello Latasa, Rosa Sancho, Jacobo Mendioroz, Montserrat Martinez-Marcos, Enriqueta Muñoz Platón, María Victoria García Rivera, Olaia Pérez-Martinez, Rosa Álvarez-Gil, Eva Rivas Wagner, Nieves López Gonzalez-Coviella, Matilde Zornoza, M Isabel Barranco, M del Carmen Pacheco, Virginia Álvarez Río, Miguel Fiol Jaume, Roxana Morey Arance, Begoña Adiego Sancho, Manuel Mendez Diaz, Noa Batalla, Cristina Andreu, Jesús Castilla, Manuel García Cenoz, Ana Fernández Ibáñez, Marta Huerta Huerta, Ana Carmen Ibáñez Pérez, Belén Berradre Sáenz, Joaquín Lamas, Luisa Hermoso, Susana Casado Cobo, Manuel Galán Cuesta, Sara Montenegro, María Domínguez, Inmaculada Jarrín, Aurora Limia, Inés del Ramo Torreblanca, Ana Lameiras Azevedo, Irene Morales Arjona, Alejandra López Zambrano, M Dolores Lasheras Carbajo, José Francisco Barbas del Buey, Mª Jesús Rodríguez Recio, Ermengol Coma, Luca Basile, María Ángeles Rafael de la Cruz López, Emma Corraliza Infanzón, María-Isolina Santiago-Pérez, María-Teresa Otero-Barrós, Jaime Jesús Pérez Martín, Alonso Sánchez Migallón, Giselle Pérez Suarez, Leticia Bravo Muñoz, Itziar Casado, Guillermo Ezpeleta, Pilar Alonso Vigil, Mario Margolles, Eva Martínez Ochoa, María Merino Díaz, Julián Manuel Domínguez Fernández, Ninoska Lopez Berrios, María Victoria Jiménez Cabanillas, Daniel Castrillejo, Gorka Loroño Ortiz, Koldo López Guridi, Luis Viloria

**Affiliations:** 1 National Centre of Epidemiology, Institute of Health Carlos III, Madrid, Spain; 2 CIBER in Epidemiology and Public Health (CIBERESP), Madrid, Spain; 3 CIBER on Infectious Diseases (CIBERINFEC), Madrid, Spain; 4 The individuals are listed under collaborators.

**Keywords:** respiratory syncytial virus, hospitalisation, nirsevimab, impact, observational study

## Abstract

Using real-life data from Spain between October 2023 and March 2024, the number needed to immunise (NNI) with nirsevimab and the cost to prevent one RSV hospitalisation were estimated at 90 infants (95% CI: 77–108) and 19,700 EUR for catch-up immunisation, and 41 infants (95% CI: 35–50) and 9,000 EUR for at-birth immunisation. By month of birth, NNI and cost were lowest in infants born shortly before the RSV epidemic peak, with impact decreasing gradually for earlier or later births.

In autumn 2023, Spain recommended immunisation against respiratory syncytial virus (RSV) with the monoclonal antibody nirsevimab to all infants born between 1 April 2023 and 31 March 2024. Uptake was very high, reaching 90% coverage [1]. Impact estimates are essential to inform decision-making on population-level immunisation strategies. Here, we aimed to estimate the number needed to immunise (NNI) with nirsevimab to prevent one RSV hospitalisation and the cost per averted RSV hospitalisation using real-life data from Spain between October 2023 and March 2024, overall and by month of birth.

## Baseline risk of RSV hospitalisation in non-immunised children

We have previously estimated nirsevimab effectiveness against hospitalisation for RSV infection in children targeted for the 2023/24 nirsevimab immunisation campaign in Spain by applying target trial emulation methods to a density sample of cases and controls from the source newborn population [2]. Here, we collected the total number of eligible births in this source population and, for one region that only selected a subset of all eligible cases, we also gathered the total number of RSV hospitalisations (Table). Per-protocol estimates of nirsevimab immunisation coverage among sampled cases and controls (Table) were obtained from the density case–control study [2]. Combining both sources of information, we estimated the risk of RSV hospitalisation among non-immunised children by applying one minus the per-protocol immunisation coverage in selected cases to total RSV hospitalisations in the numerator, and one minus the per-protocol immunisation coverage in controls to total births in the denominator.

**Table:** Number of RSV hospitalisations (n = 1,131) and births (n = 200,666) in the source population and nirsevimab immunisation coverage and effectiveness in the density case–control study by month of birth, Spain, October 2023–March 2024

Month of birth	Source population^a^		Density case–control study [2]^b^			
	RSV hospitalisations (n)	Births (n)	Immunisation coverage in cases (%)	Immunisation coverage in controls (%)	Immunisation effectiveness	
					%	95% CI
**Catch-up immunisation**	**504**	**103,372**	**51.7**	**83.0**	**80.3**	**75.3–84.4**
April 2023	46	16,260	54.3	76.2	65.3	31.6–82.4
May 2023	62	16,771	45.1	77.3	78.4	59.9–88.4
June 2023	60	17,204	50.0	86.7	85.3	71.0–92.5
July 2023	93	18,057	47.4	83.9	85.6	73.9–92.1
August 2023	99	18,470	51.2	85.1	83.5	72.1–90.2
September 2023	144	16,610	57.5	83.8	76.4	63.5–84.7
**At-birth immunisation**	**627**	**97,294**	**72.5**	**94.0**	**83.1**	**78.5**–**86.8**
October 2023	231	20,317	75.4	93.5	79.7	69.3–86.6
November 2023	242	19,363	68.4	94.5	87.7	81.0–92.0
December 2023	117	20,038	75.2	93.1	78.3	64.9–86.6
January/February 2024	37	37,576	71.9	96.3	83.9	62.3–93.1

CI: confidence interval; RSV: respiratory syncytial virus.

^a^ The source population consisted of all eligible births from 1 April 2023 to the onset of immunisation campaigns (primarily between 25 September and 6 October 2023) for catch-up immunisation and from campaign onset to 29 February 2024 for at-birth immunisation in the whole public hospital network of 14 Spanish regions (Andalusia, Asturias, Basque Country, Cantabria, Castile and Leon, Castilla-La Mancha, Ceuta, Galicia, La Rioja, Madrid, Melilla, Murcia, Navarre and Valencia) and in selected major public hospitals of the remaining five regions (Aragon, Balearic Islands, Canary Islands, Catalonia and Extremadura). Five regions were excluded from catch-up immunisation because their campaigns began in late October or November (Balearic Islands, Basque Country, Extremadura and Melilla) or were not implemented (Navarre).

^b^ Results from the density case–control study have been previously published [2]. Briefly, this study included all RSV hospitalisations in the source population for all Spanish regions except Andalusia (which selected a subset of all cases) and four density (risk-set sampled) controls per case matched on province and birthdate (± 2 days). Causal per-protocol estimates of immunisation coverage and effectiveness were obtained by creating two clones per selected case/control children, assigning each clone to either immunisation or no immunisation with nirsevimab (in the first 30 days of campaign for catch-up immunisation or in the first 14 days of life for at-birth immunisation), and censoring clones who deviated from their assigned immunisation. Effectiveness was estimated as one minus the causal rate ratios obtained from inverse-probability-of-censoring weighted conditional logistic regression models.

Overall, the risk of RSV hospitalisation was 13.8 (95% confidence interval (CI): 12.2–15.7) cases per 1,000 non-immunised children targeted for catch-up immunisation and 29.4 (95% CI: 25.3–34.1) cases per 1,000 non-immunised children targeted for at-birth immunisation. By month of birth (Figure), the risk of RSV hospitalisation among non-immunised infants increased progressively from 5 cases per 1,000 in infants born in April 2023 to 72 cases per 1,000 in those born in November 2023 and then decreased to 7 cases per 1,000 in infants born in January or February 2024. The RSV epidemic peak in the 2023–24 season in Spain occurred in mid-December and, because the risk of severe RSV declines drastically in the first few months, this risk gradient by month of birth was expected and is consistent with findings from published cohort studies [3,4].

**Figure:**
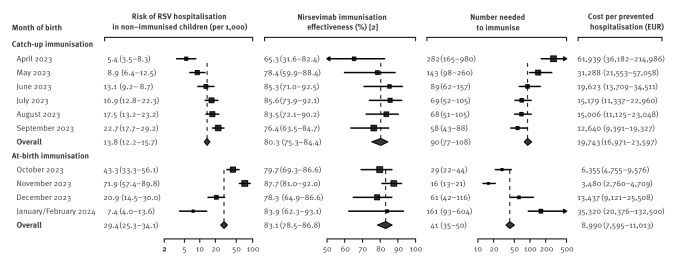
Number of children needed to immunise with nirsevimab to prevent one hospitalisation for respiratory syncytial virus infection and cost per prevented hospitalisation by month of birth, Spain, October 2023–March 2024

RSV: respiratory syncytial virus.

The area of each square is inversely proportional to the variance. Horizontal lines represent 95% confidence intervals. Nirsevimab immunisation effectiveness as estimated in causal per-protocol analysis in Núñez et al. [2].

## Number needed to immunise and cost per prevented RSV hospitalisation

Per-protocol effectiveness was obtained from the previous case-control study [2], estimated at 80% for catch-up immunisation in infants entering their first RSV season and at 83% for at-birth immunisation in children born during the RSV season (Table), which were consistent with efficacy estimates of 77% to 83% in clinical trials [5-7]. The NNI was calculated as the inverse of the product of this per-protocol immunisation effectiveness and the above estimated RSV hospitalisation risk among non-immunised children in the source population [8,9]. The CI for NNI was derived using delta methods, which accounted for the uncertainty and correlation in both estimates. Additional methodological details are provided in the Supplementary Materials.

The overall NNI was 90 (95% CI: 77–108) for catch-up immunisation and 41 (95% CI: 35–50) for at-birth immunisation. Using a price per dose of 219.42 EUR, which included administration materials but not programmatic expenses, the cost per prevented RSV hospitalisation (NNI multiplied by the cost of immunising one child) was 19,700 EUR (95% CI: 17,000–23,600) for catch-up immunisation and 9,000 EUR (95% CI: 7,600–11,000) for at-birth immunisation. The NNI and costs varied largely by month of birth, mainly because of the observed variation in RSV hospitalisation risk in the non-immunised, as nirsevimab effectiveness remained fairly similar (Figure). The lowest NNI was 16 (95% CI: 13–21) in children born in November 2023, increasing to 282 (95% CI: 165–980) and 161 (95% CI: 93–604) in those born in April 2023 and January/February 2024, respectively.

## Discussion

The overall NNI to prevent one RSV hospitalisation has been estimated at 128 in a simulation study in the United States [10]. In the MELODY trial, for the secondary end point of hospitalisation for RSV-associated lower respiratory tract infection, 14.7 cases were averted for every 1,000 infants immunised [11], which would correspond to an NNI of 68. Discrepancies may be influenced by different baseline risk and/or age distribution among study participants, given the great variations in the NNI by month of birth found in our study.

To our knowledge, estimates of impact, such as NNI and cost per prevented hospitalisation, had not been previously reported by month of birth. One key question in the design of nirsevimab immunisation strategies is which birth cohorts to include, with practice being heterogeneous across and within countries [2,3,12]. Our results show that the impact of nirsevimab in preventing RSV hospitalisations decreases gradually for earlier birth cohorts, but the impact remains substantial in children born up to 6 months before the start of the RSV season, granting consideration of catch-up immunisation for these children.

Limitations of the case–control study have been previously discussed [2]. In addition, imprecise ascertainment of the total number of cases and/or births may have biased the estimated baseline risk in the source population. We included only cases with RSV infection confirmed by PCR, while cases diagnosed by rapid antigenic testing were excluded, which may result in an under-ascertainment of RSV hospitalisations, albeit of small magnitude, since PCR use is widespread.

Generalisability of our findings may depend on the epidemiological context and the programmatic implementation of the nirsevimab immunisation, particularly the cost per dose, which can vary greatly between countries [13]. For example, simulation studies in the United States used 445 USD/dose, estimating 19,909 USD per RSV hospitalisation averted after deducting savings of not using palivizumab, another monoclonal antibody previously used in high-risk children [10]. Importantly, we have only estimated the cost for the immunisation programme to prevent one RSV hospitalisation, but we did not account for any other costs or savings, in particular, the cost of an RSV hospitalisation. Moreover, we did not attempt to provide more comparable cost-effectiveness parameters, such as quality-adjusted life years, as found elsewhere in the literature [10,14,15]. Full-scale cost-effectiveness studies are needed that consider all relevant costs and include the full spectrum of RSV disease, from mild bronchiolitis to intensive care admissions, death and, ideally, sequelae of RSV infections. Our results may be used to refine such estimates, which should consider incorporating separate analyses by month of birth.

## Conclusion

Our results show a very high impact of the population-level immunisation campaign with nirsevimab, with one RSV hospitalisation averted for every 90 and 41 immunised infants in the catch-up and at-birth immunisation target groups, respectively. Impact was greatest with the immunisation of children born shortly before the epidemic peak. This provides key information to guide the implementation of future campaigns.

## Supplementary Data

473000 Supplement
