# Supplementary material for: Infants needed to immunise with nirsevimab to prevent one RSV hospitalisation, Spain, 2023/24 season
Source: Euro Surveill. 2025 Feb 13;30(6):2500040. doi: 10.2807/1560-7917.ES.2025.30.6.2500040 (PMC11914966; doi:10.2807/1560-7917.ES.2025.30.6.2500040)
Supplement: Supplement [file 25-00040_MONGE_Supplement.pdf]

SUPPLEMENTARY MATERIAL

Supplement to: Pastor-Barriuso R, Núñez O, Monge S, on behalf of the Nirsevimab Effectiveness Study Group. Infants needed to immunise with nirsevimab to prevent one RSV hospitalisation, Spain, 2023/24 season. Euro Surveill. 2025.

This supplementary material is hosted by *Eurosurveillance* as supporting information alongside the above mentioned article on behalf of the authors who remain responsible for the accuracy and appropriateness of the content. The same standards for ethics, copyright, attributions and permissions as for the article apply. *Eurosurveillance* is not responsible for the maintenance of any links or email addresses provided therein.

Table of contents

Supplementary Methods 2

Supplementary References 3

Supplementary Methods

An adjusted estimate of the number of children needed to immunise with nirsevimab NNI_a to prevent one hospital admission for respiratory syncytial virus (RSV) infection was obtained as

$$NNI_a = \frac{1}{R_0 - R_{1a}} = \frac{1}{R_0(1 - RR_a)},$$

where R_0 was the crude risk of hospitalisation for RSV infection among non-immunised children and $RR_a = R_{1a}/R_0$ was the risk ratio of immunised to non-immunised children standardized to the distribution of confounding factors in the non-immunised group (1, 2). Assuming a low risk of RSV hospitalisation during the epidemic season and a homogeneous risk ratio across strata of confounding factors, RR_a was unbiasedly estimated by the causal rate ratio obtained from fitting an inverse-probability-of-immunisation weighted conditional logistic model to density (risk-set sampled) case-control data (3). The non-immunised hospitalisation risk was derived as $R_0 = a_0/n_0 = a(1 - P_{\text{ca}})/\{n(1 - P_{\text{co}})\}$, where a and n were the total number of RSV hospitalisations and births in the underlying cohort, respectively, and P_{ca} and P_{co} were the immunisation coverages among sampled cases and controls. A confidence interval for NNI_a was calculated by inverting and exchanging the confidence limits for the adjusted risk difference (denominator of NNI_a) (1, 2), whose variance can be approximated by delta methods as

$$\begin{aligned}\text{var}\{R_0(1 - RR_a)\} &\approx (1 - RR_a)^2\text{var}(R_0) + R_0^2 RR_a^2 \text{var}\{\log(RR_a)\} \\ &\quad - 2R_0 RR_a(1 - RR_a)\text{cov}\{R_0, \log(RR_a)\} \\ &\approx \frac{R_0(1 - RR_a)^2}{n_0} + R_0^2 RR_a^2 \text{var}\{\log(RR_a)\} + \frac{2R_0 RR_a(1 - RR_a)}{n_0} \\ &= \frac{R_0}{n_0} + R_0^2 RR_a^2 \left(\text{var}\{\log(RR_a)\} - \frac{1}{n_0 R_0}\right),\end{aligned}$$

where the robust variance of the log-transformed causal rate ratio was obtained from the weighted conditional logistic regression. Contrary to previous approaches in case-control

studies (4), this confidence interval accounted for the uncertainty and correlation of both RR_a and R_0 (Poisson model for non-immunised hospitalisations).

Supplementary References

1. Bender R, Blettner M. Calculating the "number needed to be exposed" with adjustment for confounding variables in epidemiological studies. J Clin Epidemiol. 2002;55(5):525-30. http://dx.doi.org/10.1016/S0895-4356(01)00510-8 PMID:12007557
2. Bender R, Kuss O, Hildebrandt M, Gehrmann U. Estimating adjusted NNT measures in logistic regression analysis. Stat Med. 2007;26(30):5586-95. http://dx.doi.org/10.1002/sim.3061 PMID:17879268
3. Núñez O, Olmedo C, Moreno-Pérez D, Lorusso N, Fernández Martínez S, Pastor Villalba PE, et al. Effectiveness of catch-up and at-birth nirsevimab immunisation against RSV hospital admission in the first year of life: a population-based case-control study, Spain, 2023/24 season. Euro Surveill. 2025 [In press].
4. Bjerre LM, LeLorier J. Expressing the magnitude of adverse effects in case-control studies: "the number of patients needed to be treated for one additional patient to be harmed". BMJ. 2000;320(7233):503-6. http://dx.doi.org/10.1136/bmj.320.7233.503 PMID:10678870
